# Unfolding Hidden Narratives: Glimpses of an Ethos of Senses in a Dementia‐Care Facility

**DOI:** 10.1002/hast.4990

**Published:** 2025-09-18

**Authors:** Els van Wijngaarden

**Keywords:** dementia‐care facilities, meaningful communication, otherness, responsiveness, ethos of senses, clinical ethics

## Abstract

In this essay, I explore the possibility of meaningful communication and genuine connection within dementia‐care settings, and particularly among those living with advanced dementia. Drawing on empirical research and informed by the phenomenology of Bernhard Waldenfels, I aim to challenge dominant cultural narratives that reduce dementia‐care institutions to places of confinement and loss. I propose an approach that affirms the irreducible otherness of every human being, including persons with dementia. Through case studies and philosophical reflection, I argue that recognizing the strangeness of both the other and the self can open up mutual spaces of encounter. This, in turn, may cultivate an *ethos of the senses*: a moral attitude rooted in bodily responsiveness, in which all senses are engaged. Such a responsive posture may not only foster more humane and reciprocal relationships within dementia care contexts but also contribute to richer, more nuanced public narratives about what it means to live with dementia.

A dementia‐care facility for people living with advanced dementia represents one of the most feared places in Western society, associated with dreadful images of cognitive decline and loss of self. In popular media, long‐term‐care institutions are often portrayed as confining buildings where people are locked behind closed doors, just waiting for death to come. Focused on negativity and otherness, this depiction in cultural narratives has overshadowed the more nuanced everyday stories of people living with dementia. Without romanticizing the condition or trivializing the suffering and the feeling of alienation associated with it, I seek to explore the possibility of genuine human connection in the context of dementia units.

To this end, I draw on some of my previously published empirical research and make use of three cases extracted from two datasets of ongoing studies. I bring these empirical investigations in dialogue with the phenomenology of the German philosopher Bernhard Waldenfels, whose work examines the alien and unfamiliar in human experience, exploring notions of otherness, strangeness, and responsiveness. Next, I reflect on the question of how we can unfold hidden narratives to restore the “voices” of people living with dementia while acknowledging the feelings of otherness and strangeness that others might experience in interacting with them. By “hidden narratives,” I mean fragmented, nondiscursive narratives beyond language, speech, or cognition, residing in gestures, silences, and subtle cues. These stories encourage us to listen in new ways, to notice what might otherwise remain unseen and fade into obscurity. I will argue that, even when a person with advanced dementia may be unable to coherently narrate their own experience, we may search for meaningful encounters in which personal narratives may be explicated by those around and in which moments of responsiveness may be cultivated. Such encounters may also contribute to foregrounding and repairing more nuanced cultural narratives about dementia. I will show how this work could foster an *ethos of senses*, an idea I will elaborate on later in the essay.

## The Strange World behind Closed Doors

To counter the negative images of living with dementia and make room for more nuanced ones, it is indispensable to first try to better understand the fear evoked by the strangeness that people often experience in an encounter with a person with advanced dementia. I will set the tone by describing one of my recent research experiences, which shows how life unfolds in the often‐unknown environment where institutionalized care takes place and how that space itself already may create uncomfortable feelings for visitors.

In the beginning of 2023, I spent two weeks conducting a study in a closed unit for people with advanced dementia and severe behavioral challenges who require specialized care and support. To enter this unit, I had to walk for several minutes through a series of corridors in a huge institutional building housing several offices and departments. The unit was located on the first floor. On my way to the unit, I passed several opaque sliding doors that could be unlocked only with a code or a card. Next to each door, a sign cautioned passersby not to let residents pass through these doors. Even though I had visited such units often, I still felt a sense of alienation when the unit's entrance door closed behind me. It was as if I had walked into a world of its own, closed off from the outside world.

For hours I sat there, in the living room, listening to the sounds of the unit that quite often seemed unfamiliar and strange to me: Some residents spoke with soft, almost inaudible voices. Others used some recognizable words but repetitively and somewhat incoherently. Others articulated completely incomprehensible words, or just made noises, sometimes very loudly, like shrieks or screams. With a few residents, I could have a conversation according to what I conceive of as normal social standards, although they seemed to have forgotten about our conversation as soon as it was over.

Altogether, for outsiders, a dementia care facility may appear as an unfamiliar world, with people who seem to live, think, and feel completely differently from those on the outside. Such a facility represents an uncanny and strange world that confronts and scares people.

Consider the story of Eileen, aged eighty‐three, whom I interviewed outside the unit, for a different study. Although she was still healthy according to her family doctor and had not been diagnosed with any memory or cognitive impairment, Eileen was terrified by the idea that she was in the early stages of dementia. She told me, “As soon as I forget something, I panic. People around me try to reassure me: ‘Yeah, that's part of getting old; we all forget things.’ But I'm sure their case is not as bad as mine.” She had been evaluated for dementia several times at her own request, with all tests negative, yet Eileen did not trust the results. Every time she forgot something, her forgetfulness confirmed for her that she was indeed ill. She explained to me, “It makes me very unhappy. I have experienced the whole trajectory with my husband. He passed away from dementia a year ago. Truly, it was a horror. A failure as a human being. From a strong, active, sporty guy, he degraded into a pathetic man who eventually had to be helped with everything. As he sat there in that unit, withering away, it was such a failure. He no longer participated in conversations. He just stared at me with those empty eyes.” Eileen found it both revolting and profoundly humiliating to see her husband in such a state, and she wanted to prevent her life from ending like his. “I absolutely don't want to experience that!” she stated. Her experiences in the dementia care facility fueled her deep fears of dementia. As a consequence, she desperately sought ways to avoid dementia and living in a care unit herself.

Eileen's story is situated in contemporary Dutch society, where the public discourse around aging and dying is predominantly construed by the idea of choice and control.^1^ A widespread underlying idea is that individuals should have the possibility to live and die according to their own motives, on their own terms. The last phase of old age is often represented as a “bugaboo,” and dementia is portrayed as “the looming spectre for every human being.”^2^ In fact, it appears that some older people internalize these aging anxieties and provoke a profound negative outlook on life, even if they themselves are not in a stage of dementia.^3^ For Eileen, as for others, fear may stem from a combination of factors: the influence of the one‐sided public debate, on the one hand, and first‐hand experience as a close caregiver for someone who has or died from dementia, on the other.

The Dutch increasingly discuss the diagnosis of dementia as a potential reason to opt for a preemptive death in the form of euthanasia, specifically, an injection of a lethal dose of medication by a physician.^4^ Under Dutch law, strict criteria are applied to assess each euthanasia request. Physicians must be sure that the person has made a voluntary and carefully considered request and that the person's suffering is unbearable, hopeless, and without prospect of improvement.^5^

In early‐stage dementia, in which individuals may still be decisionally competent in relation to their euthanasia request, unbearable and hopeless suffering may stem largely from fears of further decline and the associated loss of autonomy and dignity. The Dutch Euthanasia Code acknowledges that the anticipation of progressive loss of personality, functions, and skills, coupled with the inevitability of this decline, can cause profound suffering in the present.^6^ In such cases, it is legally permissible for physicians to assess whether a patient meets all criteria for euthanasia and, if so, to perform it.

In advanced dementia, in which individuals can no longer express their will or make competent decisions, euthanasia may still be an option in the Netherlands based on an advance directive. These directives outline situations that the person had previously deemed unbearable and hopeless and have a legal weight similar to that of an oral request.

However, most Dutch physicians find such cases particularly complex. Predicting future preferences can be challenging, patients may forget about their directives, and the priorities of individuals with advanced dementia may differ significantly from those they held earlier, posing the “then‐self versus now‐self dilemma.”^7^ Consequently, there is widespread hesitation among physicians to perform euthanasia in advanced dementia, making such cases highly exceptional.^8^

Nevertheless, the legal option to choose the timing and manner of one's death has an undeniable impact on public narratives about living with dementia. In cases of early‐stage dementia, suffering may, as mentioned, stem partly from fear of decline, particularly regarding the experienced loss of autonomy and dignity. As a colleague and I have described,^9^ this suffering in response to a feared future may explain why Eileen so urgently sought the very diagnosis she dreaded, since it would give her the opportunity to request euthanasia.

Eileen's story resonates with a cultural narrative that may have currency particularly in the Netherlands, given the Dutch experience of its euthanasia law, though somewhat similar narratives are prevalent in Belgium, which also permits people with dementia to receive euthanasia, and in Canada, where legal provisions for medical aid in dying (primarily referring to euthanasia) may, depending on the province, include people with dementia who retain decision‐making capacity.^10^

In the much more restrictive U.S. context, where medical aid in dying (involving a self‐administered dose of lethal medication prescribed by a physician) is legal in ten states plus Washington, DC, tighter eligibility criteria in effect rule out access by people with dementia. There is ongoing discussion and debate in the United States about whether a person facing dementia should be able to hasten their own death in ways that involve medical personnel.^11^ This level of interest suggests that choosing death is more widely considered as a way to safeguard oneself from the uncomfortable sense of losing control, a life of dependence, and the shame that is widely associated with deep dementia. Together with a prevailing discourse that holds up healthy, active, and successful aging as the ideal for older adults, the idea of a self‐chosen death as a reasonable way to protect oneself from anticipated perils may exacerbate the negative image of actually living with advanced dementia and may therefore widen the gap between those who live with the condition and those who do not.

## A Phenomenology of Otherness

Why do people feel such profound discomfort at the idea of living with dementia? Scholars have explored how societal perceptions of aging are shaped by negativity and a sense of “otherness,” with dementia amplifying these fears and contributing to an even more stigmatized and unsettling image.^12^ This negativity stems from a culture that privileges rationality and views cognitive abilities as the bedrock of identity, personhood, and relationships.^13^ Within this paradigm, individuals with dementia who are unable to meet these societal expectations of rationality tend to be marginalized and treated as less than fully human.^14^

The philosophy of Bernhard Waldenfels^15^ can deepen our understanding of these cultural narratives surrounding dementia and the fears they evoke. His phenomenology of otherness illuminates the relational dynamics underlying our resistance to dementia and provides a framework to explore why people like Eileen, and probably also ourselves, experience distress when confronted with (the prospect of) dementia.

Waldenfels emphasizes that every human being remains, in an essential sense, inaccessible and unknowable to others. While empathy plays a critical role in our interactions, it has limits: we can never fully see the world as another does or completely share their experiences. But the unknowability of the other, Waldenfels argues, is not a deficiency. It is a foundational ethical point. It is precisely this irreducible otherness that constitutes the unique subjectivity of each individual and demands our respect.

Yet profound otherness can also evoke confusion and unease. Encounters with the unfamiliar—or otherness—challenge the self‐evidence of our world and disrupt what we take for granted. These encounters may provoke the unsettling realization that life resists our attempts to control or fully comprehend it. When faced with such otherness, Waldenfels^16^ observes, we tend to resist, distance ourselves from, or attempt to assimilate it. Such responses aim to “prevent the alien from disturbing us from within,” providing a sort of “safety device … seeking a hold in what is common.”^17^ We seek to suppress what feels alien by framing it within the confines of our own familiarity.

While this tendency is present in all human encounters, it becomes especially pronounced in interactions where the “otherness” of another person is starkly apparent. A care facility for people living with advanced dementia, for instance, can confront us with a world that feels entirely alien: a disorienting environment, inaccessible emotions, unfamiliar behaviors, and strange language or gestures. Such encounters may provoke a subconscious desire to distance ourselves from this strangeness, leading to denial, neglect, or rejection or causing one to look away.

To move beyond these reactions, Waldenfels argues, we must recognize the inherently relational nature of otherness. Encounters with the unfamiliar are not one‐sided. Rather, they are an intersubjective enterprise, involving “us” as much as “them.” Strangeness is not a characteristic of the other person; it arises in the relational space between us. In this sense, the unsettling nature of dementia does not merely reflect the other's difference but also reveals something about us—our fears, assumptions, and vulnerabilities.

By applying Waldenfels's insights, we can reframe the question of how to move beyond the stigmatizing images of dementia into a relational inquiry, which entails a two‐way thinking. Engaging with the experience of otherness requires us to reflect on our pre‐reflective reactions and how these shape our interactions. Such self‐reflection can deepen our understanding not only of the other (whom we may perceive as alien) but also of ourselves. This process may challenge the boundaries of our own worldview and open up new perspectives, enabling us to ask valuable questions: Why does dementia make me so uncomfortable? What does my discomfort reveal about my desires, fears, and assumptions about humanity? Ultimately, Waldenfels's phenomenology encourages us to embrace the relational and transformative potential of encounters with otherness. By doing so, we may find ways to foster meaningful connections with individuals living with dementia, moving beyond unease toward a more inclusive understanding of humanity.

## The Former Director and the Pigsty

Maurice, aged eighty‐two and a former director of a large company, had been residing in this unit for a few months. He was slender, neat, and stylishly dressed. He often spent time in the shared living room. Every now and then, he took a comb out of the back pocket of his trousers and ran it through his hair. “Each morning a little pomade, and it looks fine for the rest of the day.” He usually carried a black notebook with him, and throughout the day, he wrote down all kinds of observations. “Then I know exactly what happened,” he stated, as if the practice gave him some kind of grip on his situation. “Do you want to see my room?” he asked when we were having coffee in the living room.

When we entered his room, he seemed startled and began to apologize awkwardly. “Oh, sorry, don't look at this.” He pointed to a so‐called posey bed, a kind of tent bed that can be zipped up completely. It is intended to enclose someone who is restless or exhibits erratic behavior and to support a calming effect. But Maurice had the greatest difficulty in relating to the bed. His aversion seemed to be reinforced now that I was standing next to him. He told me that he found it hideous, demeaning, and deeply shameful. He said, “Sorry, it looks like a pigsty.” He looked at me for a moment and then firmly turned his back to the bed. He preferred to point to his photos on the wall that pictured his previous sailing ship, his car, and his motorcycle. He was also proud of his former office desk, telling me, “It was cut to size especially for here; my wife put it here.”

I reflected upon my interaction with Maurice for a long time afterward. What exactly had happened here? What did the posey bed represent, for Maurice but also surely for me? Why did I feel uncomfortable and uneasy? And how did my gaze shape the moment? Because, to be honest, the posey bed had at least momentarily shocked me. It was the first time I had seen such a bed in someone's room: a large—and, in my view, quite ugly—object. I also found it quite disturbing that a grown man had to sleep in such a closed space, and I had not expected that Maurice was in need of such a drastic measure.

At the same time, I sensed Maurice's confusion and uneasiness. Without doubt, for a moment he felt gazed at. His comb, the notebook, the photos of his car and sailboat, and his desk are living memories connecting him to his past. They symbolically maintain his identity, giving him dignity and pride. Indeed, the photos evoke a sense of freedom, travel, and movement that contrasts sharply with living in a closed unit and having to sleep in a posey bed. In fact, the posey bed represented his deterioration and the loss of autonomy, symbolizing his otherness. I noticed that he seemed to be scrutinizing my recording gaze. I didn't know how and where to look. I stared fixedly at the bed, but I felt an urge to look away, as if nothing was at stake. I wanted to look open, attentive, and accepting, but for a moment I didn't know how.

Waldenfels sheds light on such moments. “The alien awakens my attention or just my curiosity and as such it brings about a disturbance of attention,” he observes.^18^ Pre‐reflexively, our gaze, before we become aware of it, creates a moment where the inclusion of the other seems to fail. We have become voyeurs or, as Waldenfels puts it, “bothersome spectators” with a surveying gaze.^19^ Moreover, Waldenfels points out, our gazing away often also includes some gazing toward, even if it is only out of the corner of our eyes.^20^

The gaze upon another has the potential to confirm and recognize that person's being, creating a safe and accepting space, but may also appear disapproving, scrutinizing, and inquisitive, creating a sense of distancing and otherness. My moment with Maurice also shows the importance of a distinction between a response *content* and a response *event*. Waldenfels points out that responding does not necessarily begin with talking about something, is not restricted to the content of spoken utterances, but is instead about a situationally embodied event. No matter what we say or do, an event or encounter initially starts with the senses, with a looking at, a glance, with intersecting gazes. The logic of embodied responsiveness contrasts sharply with a logic based on rational understanding.^21^ Waldenfels describes it as an “ethos of the senses”^22^—a moral attitude rooted in the body, where responsiveness engages all our senses.

## Unfolding Hidden Narratives

The professional caregivers at Maurice's care unit seemed quite at ease in this world of its own. They demonstrated a certain openness to what might come about today. What may appear alien to the outside world did not seem to disturb them. In the safe space within the walls of the institution, they cultivated ways for residents to live with finitude and decline meaningfully, not dominated by the prevailing negative images. Even as life was slowly falling apart, meaningful, responsive encounters took place. The caregivers functioned as preservers of recognition; they approached the people living with dementia as recognizable and recognized persons. They were moving along, tuning in and seeking connection through small sounds, movements, gestures, and expressions. They embodied a Waldenfelsian ethos of the senses.

I saw this ethos clearly in the care provided to Bart, aged seventy‐nine, by Debora. Bart, always an early bird, was usually one of the first to have breakfast. One morning, when he was finished, Debora, a caregiver, walked toward him, bent down on her knees, put her hands on his legs, looked at him with a smile, and asked, “Bart, shall we freshen you up?”

Softly, almost inaudibly, he agreed. “Of course.” Arm in arm, they walked to his bedroom. The door was half barricaded by a large, heavy armchair. Debora calmly made room so they could enter.

She later explained to me that Bart often blocked his door. “It has to do with the past; he has lived a somewhat dissolute life, and therefore he is always a bit wary and frightened.”

Debora asked Bart to sit on the mobile commode chair in the bathroom. In most of her questions and instructions, she used his first name, as if she wanted to reinforce the memory of his name and keep him engaged: “Bart, shall we take your pants off now?” “Bart, could you help me wash your face?” “Bart, shall I dry your back?” “Bart, if I do your teeth, will you shave yourself?” And every time he cooperated, or tried to cooperate, she said, “Thank you, Bart. Together, we fix the job easily.”

Later, sitting on the edge of his bed, when putting on his socks and shoes, he pointed to one of his few photos. “Mom,” he mumbled.

Debora looked at him and at the photo and said, “Yeah, your mom was a sweetheart, wasn't she?” Meanwhile, she put on his support stockings. She rubbed his legs calmly and attentively.

Then, all of a sudden, he said that he had been “worrying” all night. No, he hadn't sleep well. Hardly intelligible, he whispered something about his father. With her voice barely above a whisper, she responded, “That wasn't fun, was it? No, we know about that.” In the meantime, she continued gently rubbing his legs. And then smoothly she brought the conversation back to his mother. Later, she told me that his father used to be overbearing and that Bart relived it sometimes during the night. When Bart was fully dressed, she thanked him again for the cooperation. Arm in arm, she walked him to the living room for a cup of coffee.

Research suggests that people living with dementia retain a desire for a sense of meaning in life.^23^ They long to feel connected and involved, and they express the importance of feeling welcomed, recognized, and accepted as a person.^24^ As dementia progresses, they might nevertheless struggle to articulate their needs and longings and to narrate their own experiences. Therefore, we may need to search for meaningful encounters in which moments of responsiveness may be cultivated. Family members and involved professional carers report a kind of responsibility in this regard; they feel that they should do something to confirm dignity and meaning in the lives of those living with dementia. Some indicate that it was mainly about “maintaining” someone's dignity, or “searching” for dignity, or “assigning” meaning and a sense of dignity to the situation by taking a loving attitude.^25^

What is more, in the midst of the struggle between holding onto and losing grip on life, caregivers like Debora seem to be able to create an accepting space for nondiscursive, unarticulated, and scrappy stories. Debora showed how to listen to those fragments, to go beyond speech and cognition and subtly elicit the hidden narratives of those living with dementia. What I observed was a mixture of crouching down, eye contact, soft touch, calmness, attentiveness, and laughter. By speaking in the first‐person plural—talking about what “we” are doing—as well as expressing gratitude, Debora nurtured a sense of togetherness and reciprocity. Witnessing and briefly naming the sad and dark pages of a person's life story (and then shifting attention) acknowledges who that person was and is and restores that person's sense of having a voice. Personhood and subjectivity are upheld.

Some family caregivers may be able to go beyond their experience of otherness and alienness, acknowledge the relational nature of this experience, and overcome the uneasiness. It may enable them to (re)enter into a responsive relationship. It is, however, important to acknowledge that, for family caregivers such as Eileen, it may be considerably more difficult to deal with the uneasiness than for professional carers such as Debora. After all, family carers are often deeply involved on an emotional level. Their feelings of alienation in relation to their relative and the environment are often linked to multiple, ongoing, and ambiguous loss experiences and sorrows.^26^ Their relationship with their loved one has deeply altered and continues to change and slip away. Absence and presence coexist. There is no clarity, no control, and no predictable journey; they do not know what is coming next and where it will end.^27^ While Debora could connect to the personal history of Bart from a professional stance, for family members, the personal history is often a shared lived history, potentially arousing sadness, grief, or anger. When a professional carer acknowledges how overwhelming the feelings of alienation and loss can be for relatives, this recognition can offer a sense of relief to them. In such cases, professionals like Debora may substitute in doing a task that is perceived as too big by family.

## Toward an Ethics of Senses

To better understand anxiety about dementia, and therefore to better care for people with dementia, fully acknowledging what challenges and frightens us may be a better starting point than rejecting our fear. Waldenfels makes a plea for engaging with the experience of the alien without “pathologizing” it, but also without “domesticating” it, and he suggests that we can easily adapt to the alien and overcome the distinction between the own and the other.^28^ To enable meaningful encounters, he argues, we should rather acknowledge the irremovable, unknowable character of the otherness of the other and keep it intact.

As we do that, we may come to see that our experiences of otherness have the potential to connect rather than divide us. The confusing encounter with the other may lead to the realization that the world is less self‐evident. It may stimulate humble, reflexive knowledge about our own partially limited views and may confront us with our own discomfort, awkwardness, urge for control, and fears. We can then approach people with dementia not from our own categories and worldviews but by being open to theirs, striving to make room for their experiences and their individuality. We may also come to realize that we ourselves are as much strangers to the other as they are strangers to us. This realization may enable us to regard the experience of otherness and alterity as an important mutual meeting space, a challenging exchange of thoughts between what is our own and what is alien to us. Hence, it might be a driver for a responsive ethical attitude that helps us to open up and connect with someone who at first may appear deeply alien. Such responding embodies an ethos of the senses that may not only contribute to more meaningful and reciprocal one‐to‐one encounters but also generate more nuanced cultural narratives about living with dementia.

## Acknowledgments

I am grateful to all the participants in the two studies whose case descriptions I used, and who generously welcomed me into their lives. Special thanks go to the anonymous reviewers for their helpful and constructive feedback on the manuscript.
